# OFD1 and VFL3/CCDC61 in basal body positioning and docking in *Paramecium*

**DOI:** 10.1186/2046-2530-4-S1-P29

**Published:** 2015-07-13

**Authors:** H Bengueddach, M Lemullois, J Cohen, AM Tassin, A Aubusson-Fleury, F Koll

**Affiliations:** 1 CGM, CNRS, Gif Sur Yvette, France

## Objectives

Ciliogenesis is conditioned by a correct positioning/anchoring of the basal body at the cell surface. In *Paramecium *we have shown that three conserved proteins FOR20, centrin 2 (CEN2) and centrin 3 (CEN3) participates in this process, with FOR20 and CEN2 being also involved in the transition zone assembly. We established a chronology in basal body assembly: CEN2 is required for FOR20 recruitment, the latter being necessary to recruit CEN3. Our goal now is to integrate others molecules in this cascade.

## Methods

We used a combination of electron microscopy, immunocytochemistry, GFP protein tagging and RNAi knockdowns to study the function of OFD1 and VFL3/CCDC61 in *Paramecium*. OFD1 is a well-studied protein which is involved in human development whose mutations in human males can impair basal body docking. In contrast, only studies in *Chlamydomonas *indicate that VFL3 could be involved in this phenomenon.

## Results

As in human, the depletion of OFD1 in *Paramecium *induces defects in basal body docking, these defects being similar to those observed upon inactivation of FOR20, CEN2 and CEN3; 1) like FOR20 and despite its distal location on anchored basal bodies, OFD1 is recruited early during their assembly; 2) while the recruitments of OFD1 and CEN2 proceed independently, the two molecules are required for the recruitment of FOR20. We also present preliminary results indicating that VFL3/CCDC61 is crucial for maintaining both basal body polarity and positioning and for the recruitment of CEN3, but neither for CEN2 or OFD1.

